# Does health insurance coverage improve cardiometabolic risk factor levels? Quasi-experimental evidence from India

**DOI:** 10.1080/16549716.2025.2570600

**Published:** 2025-10-14

**Authors:** Kavita Singh, Dimple Kondal, Meetushi Jain, Deepa Mohan, Devraj Jindal, Ruby Gupta, Vamadevan S Ajay, Viswanathan Mohan, Rajeev Sadanandan, Anubha Agarwal, KM Venkat Narayan, Nikhil Tandon, Mark D. Huffman, Mohammed K. Ali, Dorairaj Prabhakaran, Manuela De Allegri

**Affiliations:** a Centre for Chronic Conditions and Injuries, Public Health Foundation of India, New Delhi, India; b Heidelberg Institute of Global Health, Heidelberg University Hospital and Medical Faculty, Heidelberg University, Heidelberg, Germany; c Biostatistics and Data Management, Centre for Chronic Disease Control, New Delhi, India; d Department of Epidemiology, Madras Diabetes Research Foundation, Chennai, India; e Goa Institute of Management, Sanquelim Campus, Goa, India; f Health Systems Governance, Health System Transformation Platform, New Delhi, India; g Division of Cardiology and Global Health Center, and School of Public Health, Washington University, St. Louis, MO, USA; h School of Public Health, Washington University, St. Louis, MO, USA; i Rollins School of Public Health, Emory Global Diabetes Research Center of Woodruff Health Sciences Center and Emory University, Atlanta, GA, USA; j Department of Family and Preventive Medicine, School of Medicine, Emory University, Atlanta, GA, USA; k Department of Endocrinology and Metabolism, All India Institute of Medical Sciences, New Delhi, India; l The George Institute for Global Health, UNSW, Sydney, Australia

**Keywords:** Quality of Care for Chronic Conditions, Chronic conditions, insurance, cardiometabolic risk factors, quasi-experiment, propensity score

## Abstract

**Background:**

Chronic conditions cause notable health and economic burdens. While health insurance enables access to healthcare, its effects on chronic care outcomes remain under-explored.

**Objective:**

To examine the association between health insurance coverage and cardiometabolic risk factors among people with chronic conditions in India.

**Methods:**

Data from the Centre for Cardiometabolic Risk Reduction in South Asia (CARRS) and Solan studies, including 2,926 adults with chronic conditions were analyzed using propensity score weighting to evaluate the associations between health insurance and cardiometabolic risk factors (HbA1c, low-density lipoprotein cholesterol [LDLc], and blood pressure [BP]) and self-reported health status (measured using European Quality of Life Visual Analogue Scale [EQ-VAS]). Mediation analysis evaluated healthcare visits as a potential mediator.

**Results:**

Among 2,926 respondents meeting criteria, mean (SD) age was 54.6 years (11.8), and 1630 (55.7%) were women. Health insurance coverage was low (6.5%) and more prevalent among men, higher-income groups, and rural vs urban residents. Insured participants had lower mean diastolic BP (84.8 vs 86.0 mmHg), mean LDLc (113.3 vs 117.2 mg/dl), mean HbA1c (6.9% vs 7.5%), and higher health status (EQ-VAS: 74.6 vs 69.1) than uninsured participants, respectively (*p* < 0.05). Mediation analysis showed healthcare visits strongly mediated the relationship between insurance and BP and partially mediated effects on LDLc, HbA1c, and self-rated health.

**Conclusion:**

Health insurance coverage was associated with better cardiometabolic risk profiles and health status, largely mediated by increased healthcare utilization. Expanding insurance coverage to include outpatient chronic care services should be prioritized to improve health outcomes in low- and middle-income countries.

## Paper Context

- **Main findings:** Insurance coverage among people with chronic conditions in India is low, yet it is associated with better cardiometabolic risk factor levels and self-rated health, mediated by increased healthcare utilization.
- **Added knowledge:** This study provides empirical evidence on the strong association of insurance status on health outcomes using a quasi-experimental design (propensity score weighting).
- **Global health impact for policy and action:** Expanding insurance coverage to include outpatient chronic care is vital for improving chronic care quality and reducing premature chronic disease mortality.

## Background

Non-communicable diseases (NCDs) cause 43 million deaths globally each year, more than all other diseases combined [1]. More than 80% of NCD deaths now occur in low and middle-income countries (LMICs), including India, affecting relatively younger populations in their most productive years [2]. Cardiometabolic risk factors, such as elevated blood pressure (BP), poor glycemic control, and dyslipidemia, contribute substantially to this burden and often remain poorly managed due to fragmented access to health services [3–6]. The disease burdens caused by NCDs are coupled with substantial economic losses, leading to devastating social impacts on households [7]. Yet, in India and in LMICs more broadly, lack of insurance coverage for NCDs represents a growing concern [8,9]. The 2015 United Nations Sustainable Development Goal (SDG) target 3.8 [10] explicitly calls for urgent action by all countries to achieve Universal Health Coverage, with the ambition to enhance access to quality health services for all people, irrespective of their ability to pay. In reality, however, most LMICs are far from achieving this target. This is because penetration rates of existing health insurance schemes are low, but also because such schemes rarely include coverage for NCDs [11].

For instance, in India the health insurance landscape comprises a mix of public (e.g. Rashtriya Swasthya Bima Yojana (RSBY), Ayushman Bharat- Pradhan Mantri Jan Arogya Yojana [PMJAY]), private, and employer-based schemes. Most publicly funded schemes in India cover inpatient care, but often neglect outpatient services, which are critical to ensure access to quality chronic care. Moreover, despite efforts to expand coverage, around two-thirds of Indians remain uninsured [12,13]. This limited reach leaves millions of lives vulnerable to catastrophic health expenses, especially when managing chronic conditions that require long-term care. Even among insured, the scope of coverage is often inadequate, particularly for NCDs. As a result, households with NCDs continue to face high out-of-pocket expenses for routine consultations, medications, and diagnostics [14]. This fragmented coverage weakens the potential of health insurance to improve chronic care quality and prevent disease progression [15–17]. Both the lack of health insurance and inadequate coverage have been associated with reduced preventive care and service utilization among adults with chronic conditions [18–21]. In contrast, stable health insurance has been linked to higher rates of hypertension and diabetes screening and treatment [22,23].

While substantial literature examined the relationship between health insurance coverage and healthcare access, evidence on its impact on specific cardiometabolic outcomes in LMICs remains limited [24,25]. Few studies in LMICs have used quasi-experimental designs to assess these links, leaving important evidence gaps for chronic disease policy and planning [26]. This study addresses that gap by evaluating the association between health insurance status and cardiometabolic outcomes in India. Further, we performed mediation analysis to test the hypothesis that the association between insurance coverage and cardiometabolic outcomes is mediated through healthcare utilization, specifically the frequency of outpatient visits. This approach is grounded in health systems theory, which posits that financial protection can reduce cost barriers, thereby enabling consistent patient engagement and continuity of care that improve chronic disease outcomes.

## Methods

### Setting

The primary data included in this study were collected in three diverse Indian cities (Delhi – urban North India, Chennai – urban South India and Solan – rural North India), representing distinct demographic, socioeconomic, and epidemiologic profiles, enhancing the generalizability of our findings by capturing India’s dual burden of chronic disease challenges across both urban and rural settings.

Around the time of data collection, in 2011–2012, around 17% of the population were covered by any health insurance, which increased to about 24% by 2014–2015, which coincided with data collection for Solan [8,12]. Most public insurance schemes (e.g. RSBY) at the time of study data collection covered costs of hospitalizations, and offered subsidized outpatient care services at public health facilities [27,28].

### Design and participants

We obtained data from the baseline cross-sectional survey of the **C**entre for c**A**rdiometabolic **R**isk **R**eduction in **S**outh Asia (CARRS) cohort in Delhi and Chennai [29] (2010–2011). Solan study data were gathered in 2014. The detailed methodology of the CARRS [29] and Solan studies [30] have been published elsewhere [30–32]. Briefly, CARRS-1 recruited 12 270 adults aged ≥20 years using multistage cluster random sampling from Chennai and New Delhi. The rural Solan Surveillance study was a community-based household survey performed in Solan district in Himachal Pradesh in North India [30]. In this paper, we included data from non-pregnant adults aged ≥20 years with any chronic conditions, i.e. those who answered ‘yes’ to the following question: ‘Have you ever been told by a doctor that you have any of the following chronic conditions such as heart disease, stroke, diabetes, hypertension, dyslipidemia or chronic kidney disease?’ and those who reported using outpatient or inpatient care services for chronic conditions in the past 12 months.

Further, we employed a quasi-experimental design using propensity score weighting to adjust for non-random health insurance assignment. This approach approximates causal inference by balancing covariates across insured and uninsured groups.

### Data collection and measurements

We used standardized data collection tools to capture measurements at both sites in the CARRS Study (Chennai, and Delhi) and the Solan Study (Solan district, Himachal Pradesh). A detailed summary of all measures, and instruments used in the study has been published [32]. Briefly, a questionnaire was administered to collect information regarding the participant’s demographic, socioeconomic, behavioral, and past and present health status. Trained interviewers collected data through household interviews in local languages and measured anthropometric parameters (height, weight) using standardized techniques and BP twice at each participant’s home or a medical camp organized in the community after 5 minutes in a seated position using an electronic BP measuring device (Omron Dalian, Liaoning Sheng, China). A third reading was taken if the difference between the first two systolic or diastolic BP readings was more than 10 mm Hg or 5 mm Hg, respectively. Average BP of the two/three readings was recorded in the study database. In addition, a fasting venous blood sample was taken from the participants to test lipid profile, fasting plasma glucose, and glycated hemoglobin (HbA1c). In the Solan study, standardized clinical examinations and point-of-care fasting capillary blood glucose finger stick sampling were performed in participants’ homes. A subset of participants was selected using a convenience technique to undergo venous blood samples to analyze the lipid panel.

The response rates in the CARRS study were 94.7% for questionnaire completion and 84.3% for blood tests. The response rate in the Solan study was 96.3% for questionnaire completion and blood tests were done in a sub-sample.

### Outcome variables

The outcome variables selected were cardiometabolic risk factors, both continuous measures (mean BP, mean LDLc, and mean HbA1c) and binary measures defined as blood pressure: BP ≥ & < 140/90 mmHg, blood cholesterol: LDLc ≥ & < 130 mg/dl) and glycemia: HbA1c ≥ & < 8% and health status measured using validated European Quality of Life 5 Dimension Visual Analogue Scale (EQ5D-VAS); self-rated health status on a scale: 0–100.

### Exposure variables

Health insurance coverage was classified as a binary variable (‘any’ vs. ‘none’) based on participant self-report to the question: ‘How did you pay for your treatment and clinic visits?’, and ‘Did you get any reimbursement from insurance?’. Data on insurance type, scope of services covered, and depth of financial protection were not collected, which limited our ability to examine heterogeneity in effects across public versus private health insurance schemes with broader benefit packages.

### Other covariates

We measured healthcare utilization using the following: 1) the number of times the respondent had outpatient care visits during the past 12 months, based on the following question, ‘During the past 12 months, how many times have you seen a doctor or other healthcare professional about your chronic condition at a doctor’s clinic?’ we categorized outpatient healthcare utilization as three levels: none, one to three times per year, four or more times per year; 2) type of healthcare provider categorized as government, private, and charitable or others. The details of other covariates and categories: self-reported age, education attainment, occupation status, household income, asset index, body mass index (kg/m^2^), tobacco use, and out-of-pocket expenses are provided in the appendix, supplementary Table S1 [32].

### Statistical analysis

The respondent’s characteristics are reported overall, and by insurance status and presented as a number (proportion) for categorical variables and means (standard deviation, SD) for normally distributed continuous variables, and medians (interquartile range, IQR) for skewed costs data, out-of-pocket expenses, and self-reported health status scores.

For the continuous outcome variables (cardiometabolic risk factors and self-reported health status), we calculated the predicted means using multivariable linear regression model by insurance status, frequency of healthcare visits, and type of healthcare provider, adjusted for age, sex and city.

Further, we analyzed the associations between insurance status and health outcomes using both traditional multivariable logistic/linear regression models, and propensity score weighting with the goal to demonstrate a step-wise analytical approach and to maintain transparency in reporting model results. Propensity score is a method used to adjust for confounding in observational studies and has theoretical advantages over conventional regression models, but relative performance in real-world scenarios is not well characterized [33].

First, we constructed logistic regression (for categorical outcomes: cardiometabolic risk factors) and linear regression (for continuous outcome: EQ-VAS health status) models for each outcome variable. Model 1 presents crude estimates from the regression model without adjustment for any covariate. Model 2 reports the odds ratio and 95% confidence intervals (CI) estimates for insured compared to uninsured adjusting for healthcare factors, i.e. healthcare visits and type of care provider. In model 3, in addition to the covariates adjusted in model 2, we adjusted for individual level factors such as age, sex and geographic location (rural/urban).

Second, we performed the propensity score weighting analysis to minimize selection bias between insured and uninsured groups. We defined propensity score as the probability of an individual to be assigned to ‘health insurance’ given other relevant covariates such as participant’s age, sex, education, household income, and location (urban or rural). We created propensity score using the following equation [34]:

ei=Pr (Zi=1|Xi),

where Z is the exposure (insurance) variable, and X is the background covariates (age, sex, education, household income, and location). Further, to estimate causal effects of the insurance on health outcomes, utilizing all participants data from both CARRS and Solan datasets, we conducted propensity score weighted analysis. We used the propensity score to estimate the inverse probability of treatment weights (IPTW), which estimates the average treatment effect (insurance effect on health outcomes) and can be expressed mathematically as:

$$\mathrm{Average}\;\mathrm{treatment}\;\mathrm{effect}:E\left(\delta \right)=E\left(Y_{1}-Y_{0}\right)$$

where E(.) is average, Y_1_ is the potential outcome for those having treatment (enrolled in insurance), and Y_0_ represents the potential outcome for those having no treatment (not enrolled in insurance) [35]. To compensate for insufficient covariate balance, the IPTW weights were included in the logistic regression models for double adjustment of covariates between insured and uninsured groups [36–38]. Propensity score weighted model estimates were reported for each outcome, adjusted for healthcare visits, type of healthcare provider and self-reported history of chronic conditions.

To account for missing data in outcomes (systolic and diastolic blood pressure, 4.0%; HbA1c, 12.2%; and LDL cholesterol, 11.4%), the Multiple Imputation Chained Equation (MICE) approach was used [39]. The imputation models included all variables used in the main analyses, including exposures (insurance), outcomes (blood pressure, LDL cholesterol, HbA1c), and covariates (age, sex, location, type of health facility, number of clinic visits), as well as auxiliary variables potentially predictive of missingness (education, income, wealth index, disease conditions (hypertension, diabetes, hyperlipidemia)). We created 20 imputed datasets and the adequacy of the imputations was assessed through diagnostic plots and comparisons of imputed and observed distributions [40]. Similarly, each regression analysis model including the propensity score weighted specification was re-fit within every imputed dataset, with propensity scores re-estimated per dataset, and estimates were pooled using Rubin’s rules to incorporate imputation uncertainty [41,42]. Results from regression models using multiple imputed data are reported as a sensitivity analysis.

**Figure 1.**
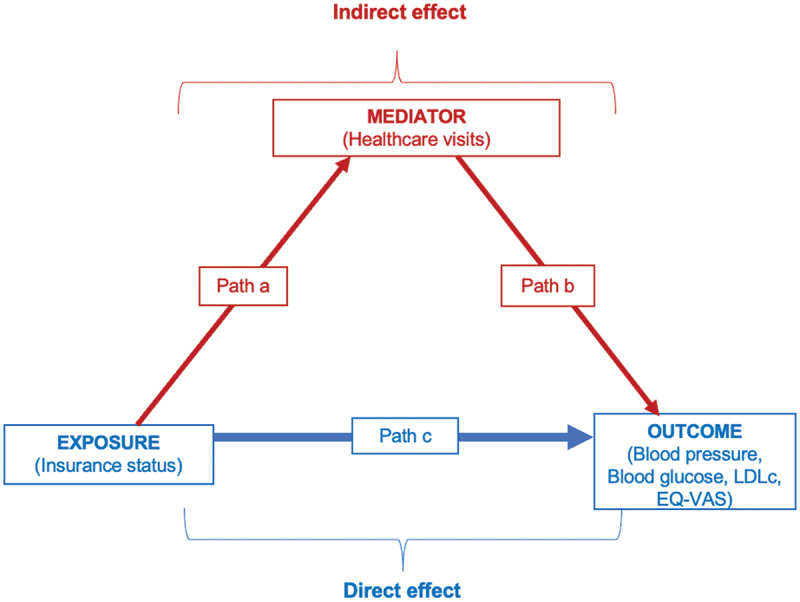
Conceptual model to guide the mediation analysis.

Lastly, we used the Baron and Kenny framework to assess whether outpatient healthcare visits mediated the effect of insurance on health outcomes (Figure 1: conceptual model to guide the mediation analysis). This approach is the most widely used method to demonstrate mediation for its conceptual clarity and stepwise procedure [43].

*The regression equations for the mediation analysis can be expressed as*:

(1) $$Y=i_{1}+\mathrm{cX}+e_{1}$$

(2) $$Y=i_{2}+c{\;}^{\prime }X+\mathrm{bM}+e_{2}$$

(3) $$M=i_{3}+\mathrm{aX}+e_{3}$$

*where c in Equation 1 defines the overall effect; c’ in Equation 2 defines the effect of the independent variable on the outcome controlling for the mediating variable and represents the direct effect in a mediation analysis; b in Equation 2 defines the effect of the mediating variable on the outcome controlling for the independent variable; a in Equation 3 defines the effect of the independent variable on the mediating variable; i*_*1*_, *i*_*2*_, *i*_*3*_ *are intercepts; e*_*1*_, *e*_*2*_, *and e*_*3*_ *are corresponding residuals*

As per Equation 1 (step 1), we regressed ‘mediating factor,’ i.e. healthcare visits on insurance status. In step 2 (Equation 2), we regressed health outcomes as continuous measures (BP, LDLc, HbA1c, EQ-VAS) on mediator (healthcare visits), and in step 3, clinical outcomes were regressed on insurance status. We used a non-parametric bootstrapping approach (with 100 replications) instead of the traditional Sobel test to estimate indirect effects and confidence intervals, as bootstrapping is more robust to violations of normality assumptions, especially in complex or smaller samples [43,44]. Further, we reported ratio of indirect to total effect (RIT), which measures the proportion of the total effect explained by mediation and the ratio of indirect to direct effect (RID), which indicates the relative strength of the indirect effect compared to the direct effect on health outcomes [45].

All analyses were performed using STATA version 18, StataCorp LLC, Texas, USA. A two-sided p-value of <0.05 was considered for statistical significance without adjustment for multiple testing.

### Ethics

The Institutional Ethics Committees (IECs) of the Public Health Foundation of India (IRB00006658), All India Institute of Medical Sciences (AIIMS), New Delhi, (IEC/NP-17/07.09.09), Madras Diabetes Research Foundation (MDRF/EC/EPI/2009/10) Chennai, India, and Emory University, USA (IRB00044159) approved CARRS study. The IECs of the Centre for Chronic Disease Control (CCDC_IEC_07_2012_amendment _01_2013) and AIIMS, New Delhi, India (IEC/NP-476/2021 & RP-19/2013) approved Solan Study. All participants provided written informed consent prior to study data collection. The research study was performed in accordance with the ethical principles stated in the Declaration of Helsinki.

## Results

### Analytical sample

**Figure 2.**
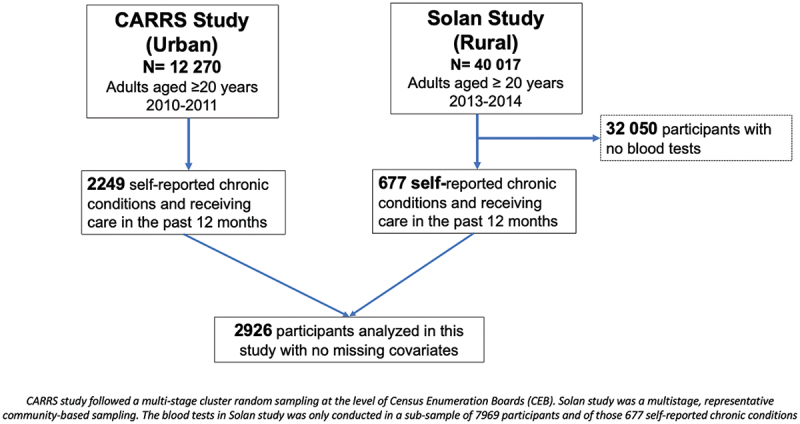
Study flow and sampling frame.

Of the 12 270 recruited participants in the CARRS baseline survey between 2010 and 2011, 2249 participants self-reported having chronic conditions and receiving care in the past 12 months. In the Solan study, of the 40 017 participants recruited over 2013–2014, blood tests were performed in a sub-sample of 7969 participants. Of these, 677 reported chronic conditions and receiving care in the past 12 months. The analytical sample comprised 2,926 participants (Figure 2). All covariates, including socio-demographic and other characteristic variables, had complete data. Self-reported health status was available for all participants. The proportions of missing data for clinical outcomes were: systolic blood pressure, 4.0%; fasting blood glucose, 12.2%; and LDL cholesterol, 11.4%.

For the sensitivity analysis, we compared baseline characteristics between participants with complete outcome data and those with missing outcome data; the two groups were similar (Supplementary Table S2).

### Characteristics of insured vs uninsured participants

**Figure 3.**
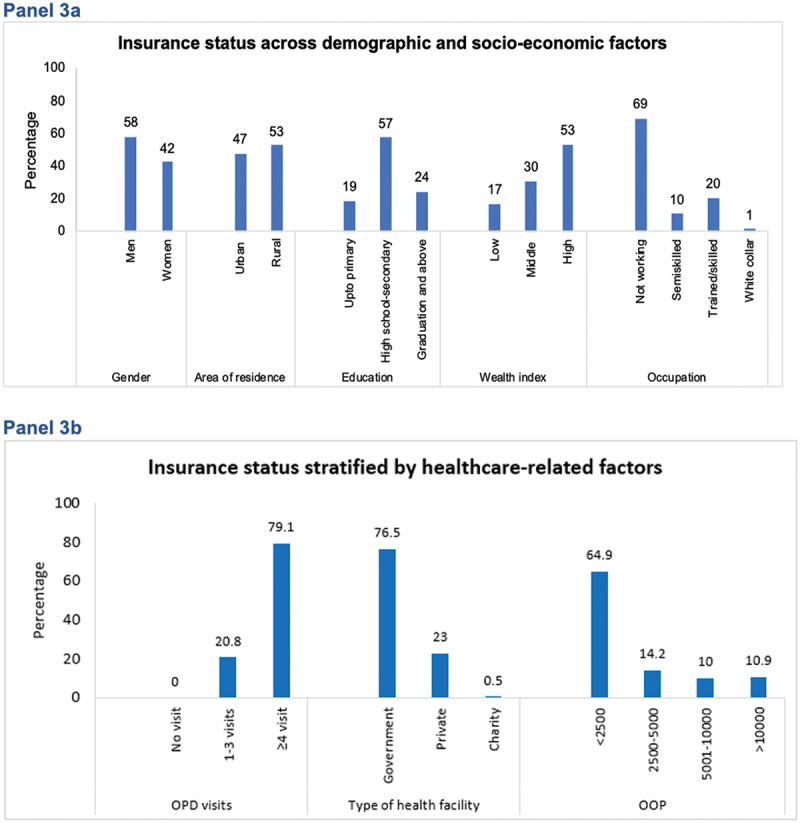
Insurance status stratified by demographic, socio-economic factors, and healthcare access.

Overall, the mean age (SD) was 54.6 (11.8) years and 57.7% were female. Around 6.5% of the study population (*n* = 190) reported having any form of health insurance. Insured people as compared to uninsured were predominantly middle-aged (mean age: 54.2 vs 59.4 years, *p* < 0.001), males (43.3% vs 57.9%, *p* < 0.001), with advanced education (graduates and above: 15.8% vs 24.2%, *p* < 0.001), greater household income (monthly household income in INR > 20,000: 22.4% vs 37.6%, *p* < 0.001), and highest tertile of asset index (43.7% vs 53.7%, *p* = 0.01, Table 1). A greater proportion of insured vs uninsured participants reported higher health status, as measured by the EQ-VAS median score (on a scale of 0–100): 70.0 vs 75.0 (*p* < 0.001), respectively. Cardiometabolic risk factors were significantly higher among uninsured compared to insured: mean (SD) BMI (kg/m^2^): 27.0 (5.2) vs 25.9 (4.5) (*p* = 0.007), mean fasting blood glucose (mg/dl) 138.6 vs 127.8 (*p* = 0.025), and HbA1c, (%): 7.5 vs 6.9 (*p* < 0.001). Further, the median annual out of pocket expenditure was greater among insured than uninsured participants (International dollars): Int$1,339 vs Int$185.3, *p* < 0.001 (Table 2). Compared to uninsured, insured people reported greater healthcare utilization defined as ≥ 4 outpatient clinic visits in the past 12 months: 61.2% vs 83.2% (*p* < 0.001) and most frequently visited public providers: 41.8% vs 83.5% (*p* < 0.001, Figures 3a, b).

**Table 1.** Demographic and socio-economic characteristics of the participants by insurance status.

	Overall N	No Insurance N (%)	Insurance N (%)	*p*-value
	2926	2736 (93.5%)	190 (6.5%)	
**Age in years, mean (SD)**	54.6 (11.8)	54.2 (11.8)	59.4 (10.9)	** <0.001**
<35 years	106 (3.6%)	104 (3.8%)	2 (1.1%)	** <0.001**
35 - 54 years	1366 (46.7%)	1307 (47.8%)	59 (31.1%)	
≥55 years	1454 (49.7%)	1325 (48.4%)	129 (67.9%)	
**Sex**				
Male	1296 (44.3%)	1186 (43.3%)	110 (57.9%)	** <0.001**
Female	1630 (55.7%)	1550 (56.7%)	80 (42.1%)	
**Education**				
Up to primary schooling	655 (22.4%)	622 (22.7%)	33 (17.4%)	**0.006**
High school to Secondary	1794 (61.3%)	1683 (61.5%)	111 (58.4%)	
Graduation and above	477 (16.3%)	431 (15.8%)	46 (24.2%)	
**Occupation**				
Not working	1963 (67.1%)	1833 (67.0%)	130 (68.4%)	0.43
Semiskilled/Unskilled	314 (10.7%)	294 (10.7%)	20 (10.5%)	
Trained/Skilled/White collar	649 (22.2%)	609 (22.3%)	40 (21.1%)	
**Marital status**				
Single	34 (1.2%)	34 (1.2%)	0 (0.0%)	0.17
Married	2554 (87.3%)	2380 (87.0%)	174 (91.6%)	
Widow/Widower	325 (11.1%)	309 (11.3%)	16 (8.4%)	
Separated/Divorced	14 (0.4%)	13 (0.5%)	0 (0.0%)	
**Income (Indian rupees, INR)**				
<10000	1486 (51.2%)	1434 (52.8%)	52 (27.5%)	** <0.001**
10,000–20000	738 (25.4%)	672 (24.8%)	66 (34.9%)	
>20000	679 (23.4%)	608 (22.4%)	71 (37.6%)	
Asset Index*				
Low	753 (25.7%)	719 (26.3%)	34 (17.9%)	**0.011**
Medium	876 (29.9%)	822 (30.1%)	54 (28.4%)	
High	1296 (44.3%)	1194 (43.7%)	102 (53.7%)	
**City**				
Chennai	1228 (42.0%)	1203 (44.0%)	25 (13.2%)	** <0.001**
Delhi	1021 (34.9%)	967 (35.3%)	54 (28.4%)	
Solan	677 (23.1%)	566 (20.7%)	111 (58.4%)	
**Location**				
Urban	2249 (76.9%)	2170 (79.3%)	79 (41.6%)	** <0.001**
Rural	677 (23.1%)	566 (20.7%)	111 (58.4%)	

*Asset index is presented in tertile, SD = standard deviation, IQR = inter-quartile range.

**Table 2.** Participants’ health status, laboratory parameters, and healthcare utilization by insurance status.

	Overall N	No Insurance N (%)	Insurance N (%)	*p*-value
	2926	2736 (93.5%)	190 (6.5%)	
**Currently use tobacco in smoke form** (Cigarette, Beedi, Cigar, Pipe)	285 (12.2%)	261 (11.7%)	24 (25.3%)	** <0.001**
**Co-morbidities**				
Coronary Artery Disease	352 (12%)	321 (11.7%)	31 (16.3%)	0.06
Hypertension	1868 (63.8%)	1750 (64.0%)	118 (62.1%)	0.61
Diabetes	1506 (51.5%)	1423 (52.0%)	83 (43.7%)	**0.026**
Chronic Kidney Disease	45 (1.5%)	38 (1.4%)	7 (3.7%)	**0.01**
Dyslipidaemia	179 (6.1%)	169 (6.2%)	10 (5.3%)	0.61
**Health status, median (IQR)**	70.0 (60.0, 80.0)	70.0 (60.0, 80.0)	75.0 (65.0, 86.0)	** <0.001**
**Health status, mean (SD)**	69.4 (16.4)	69.1 (16.4)	74.6 (15.3)	** <0.001**
**Health related quality of life (measured using EQ5D)**				
Problems in Mobility	906 (31.0%)	839 (30.7%)	67 (35.3%)	0.18
Problems in Usual activity	399 (13.6%)	368 (13.5%)	31 (16.3%)	0.27
Problems in Self-care	286 (9.8%)	271 (9.9%)	15 (7.9%)	0.37
Problems in Pain/Discomfort	875 (29.9%)	800 (29.2%)	75 (39.5%)	**0.003**
Problems in Anxiety/Depression	455 (15.6%)	416 (15.2%)	39 (20.5%)	**0.05**
**Cardiometabolic risk factors**				
**Body mass index (BMI)**, mean (SD)	26.9 (5.2) (*n* = 2480)	27.0 (5.2) (*n* = 2304)	25.9 (4.5) (*n* = 176)	**0.007**
**Systolic blood pressure (mmHg)**, mean (SD)	136 (21.3) (*n* = 2809)	135.9 (22.4) (*n* = 2621)	137.5 (19.0) (*n* = 188)	0.33
**Diastolic blood pressure (mmHg)**, mean (SD)	85.9 (12.1) (*n* = 2809)	86.0 (12.3) (*n* = 2621)	84.8 (10.1) (*n* = 188)	0.18
**Fasting blood glucose (mg/dl)**, mean (SD)	139.3 (66.2) (*n* = 2644)	138.6 (65.9) (*n* = 2466)	127.8 (55.9) (*n* = 178)	0.025
**HbA1c (%)**, mean (SD)	7.4 (2.1) (*n* = 2592)	7.5 (2.1) (*n* = 2416)	6.9 (1.7) (*n* = 176)	** <0.001**
**LDLc (mg/dl)**, mean (SD)	117.6 (37.9) (*n* = 2568)	117.8 (37.7) (*n* = 2392)	114.3 (40.1) (*n* = 176)	0.23
**Total cholesterol (mg/dl)**, mean (SD)	190.8 (43.8) (*n* = 2655)	191.2 (43.8) (*n* = 2476)	185.4 (44.4) (*n* = 179)	0.087
**HbA1c (%)**				
≥7%	1165 (44.9%)	1100 (45.5%)	65 (36.9%)	**0.027**
≥8%	811 (31.3%)	774 (32%)	37 (21%)	**0.002**
**LDLc (mg/dl)**				
≥70 mg/dl	2301 (89.6%)	2150 (89.9%)	151 (85.8%)	0.086
≥100 mg/dl	1743 (67.9%)	1631 (68.2%)	112 (63.6%)	0.21
≥130 mg/dl	918 (35.8%)	862 (36%)	56 (31.8%)	0.26
**Blood Pressure**				
≥140/90 mmHg	1331 (47.4%)	1249 (47.7%)	82 (43.6%)	0.28
**Healthcare factors**				
**No. of healthcare visits in last 12 months**				
No visit	41 (1.4%)	41 (1.5%)	0 (0.0%)	** <0.001**
1–3 visits	1052 (36.0%)	1020 (37.3%)	32 (16.8%)	
≥4 visits	1830 (62.6%)	1672 (61.2%)	158 (83.2%)	
**No. of hospitalizations in last 12 months**, mean (SD)	0.14 (0.45)	0.1 (0.4)	0.4 (0.7)	** <0.001**
**Type of healthcare facilities**				
Government	1290 (44.5%)	1133 (41.8%)	157 (83.5%)	** <0.001**
Private	1575 (54.4%)	1545 (57.1%)	30 (16.0%)	
Charitable	31 (1.1%)	30 (1.1%)	1 (0.5%)	
**Total OOP expenditure (dollars)**, median (IQR)	196.9 (26.5, 998)	185.3 (26.5, 850.8)	1339 (27.8, 4739)	** <0.001**

*SD = standard deviation, OOP = out of pocket, IQR = inter-quartile range; HbA1c = glycated haemoglobin, BP = blood pressure, LDLc = low density lipoprotein cholesterol; EQ5D = European Quality of Life 5 dimension.

Table 3 shows the predicted mean values for systolic BP, diastolic BP, LDLc, HbA1c, and EQ-VAS score representing individual’s health status by health insurance status, outpatient visits and type of healthcare provider. Compared to uninsured, insured participants had significantly lower diastolic BP (86.0 vs 84.8 mmHg), LDL-cholesterol (117.2 vs 113.3 mg/dl), HbA1c (7.5% vs 6.9%), and higher self-reported health status (69.1 vs 74.6, *p* < 0.05 for all), respectively. Participants visiting public providers had lower mean diastolic BP and HbA1c, but mean systolic BP and LDLc was lower among those visiting private providers. The overall health status was marginally higher with more healthcare visits, and with attendance at public or charitable healthcare providers.

**Table 3.** Mean cardiometabolic risk factors and health status by insurance status and healthcare factors.

	Predicted Means (95% CI)*				
Insurance status	SBP	DBP	LDL cholesterol	HbA1c	EQ-VAS
No	135.8 (135.6, 136.0)	86.0 (85.9, 86.0)	117.2 (116.8, 117.6)	7.5 (7.4, 7.5)	69.1 (68.8, 69.3)
Yes	137.4 (136.6, 138.2)	84.8 (84.6, 84.9)	113.3 (111.6, 115.0)	6.9 (6.8, 6.9)	74.6 (73.8, 75.4)
**Outpatient visits**					
No	137.7 (136.1, 139.2)	87.2 (86.8, 87.5)	119.2 (116.8, 121.6)	7.3 (7.2, 7.3)	68.8 (67.6, 70.0)
1–3	134.9 (134.6, 135.2)	86.6 (8.5, 86.7)	113.7 (113.2, 121.3)	7.6 (7.5, 7.6)	68.3 (67.9, 68.6)
≥4	136.4 (136.1, 136.7)	85.5 (85.4, 85.5)	118.7 (118.2, 119.3)	7.3 (7.3, 7.4)	70.1 (69.8, 70.4)
**Healthcare provider**					
Public	137.5 (137.2, 137.8)	85.5 (85.5, 85.6)	120.7 (120.1, 121.4)	7.2 (7.1, 7.2)	70.5 (70.1, 71.0)
Private	134.5 (134.3, 134.8)	86.2 (86.2, 86.3)	113.9 (113.5, 114.4)	7.6 (7.6, 7.7)	68.5 (68.3, 68.8)
Charitable	141.9 (140.3, 143.5)	88.5 (88.1, 88.9)	107.2 (104.2, 110.2)	7.7 (7.6, 7.8)	70.8 (68.9, 72.7)

*Predicted means calculated using multivariable linear regression model adjusted for age, sex and city.

Abbreviations: HbA1c = glycated haemoglobin, SBP = systolic blood pressure, DBP = diastolic blood pressure, LDLc = low density lipoprotein cholesterol, EQ-VAS: European quality of life – visual analogue scale (score range from 0 -100).

### Association between insurance status and health outcomes

Health insurance status was associated with a lower odds of elevated blood pressure (BP ≥ 140/90 mmHg) after adjusting for both individual and healthcare factors: adjusted OR = 0.70, 95% CI: 0.51, 0.96. Health insurance status was associated with a lower odds of elevated LDL cholesterol (LDLc ≥130 mg/dl) (adjusted OR = 0.56, [95%CI: 0.39, 0.80]), as well as in the propensity score weighted model (adjusted OR = 0.50, 95% CI: 0.31, 0.80 *p* = 0.004) (Table 4). Health insurance status was also associated with a significantly lower unadjusted odds of poor glycemia (HbA1c ≥ 8%) (OR = 0.56; 95%CI: 0.39, 0.82) and when adjusted for healthcare factors (adjusted OR = 0.65; 95% CIs: 0.44, 0.96). However, the association was attenuated when adjusted for individual and healthcare factors (adjusted OR = 0.79; 95% CI: 0.53, 1.18)) and in the propensity score weighted model (adjusted OR = 0.90; 95% CI: 0.49, 1.66), indicating potential confounding factors. Similarly, health insurance status was positively associated with higher self-reported health status in unadjusted analyses (coefficient = 5.52; 95% CI: 3.12, 7.92) and those adjusting for healthcare factors (adjusted coefficient = 4.72; 95% CI: 2.26, 7.18), but this association was attenuated (coefficient = 1.57; 95% CI: −0.78, 3.93) when adjusted for individual-level covariates (adjusted coefficient = 1.57; 95% CI: −0.78, 3.93) and in the propensity score weighted model (adjusted coefficient = 0.86; 95% CI: −2.26, 3.97). Supplementary Tables S3-S5 present detailed model results adjusting for covariates. Table S6 shows the association between health insurance status and health service utilization, adjusted for type of health provider, age, sex, and location.

**Table 4.** Association between insurance status and cardiometabolic risk factors and overall health status.

Outcomes	Model 1 (Crude)		Model 2 (adjusted for health-related factors)		Model 3 (adjusted for health-related + Individual factors)		Propensity score-weighted model	
	OR (95% CIs)	p-value	OR (95% CIs)	p-value	OR (95% CIs)	p-value	OR (95% CIs)	p-value
BP ≥ 140/90 mmHg	0.85 (0.63, 1.14)	0.28	0.80 (0.59, 1.09)	0.16	**0.70 (0.51, 0.96)**	**0.025**	0.90 (0.59, 1.37)	0.64
LDLc ≥130 mg/dl	0.83 (0.60, 1.15)	0.26	**0.63 (0.45, 0.88)**	**0.01**	**0.56 (0.39, 0.80)**	**0.001**	**0.50 (0.31, 0.80)**	**0.004**
HbA1c ≥ 8%	**0.56 (0.39, 0.82)**	**0.003**	**0.65 (0.44, 0.96)**	**0.03**	0.79 (0.53, 1.18)	0.24	0.90 (0.49, 1.66)	0.74
	Coefficient (95% CIs)	p-value	Coefficient (95% CIs)	p-value	Coefficient (95% CIs)	p-value	Coefficient (95% CIs)	p-value
EQ-VAS	**5.52 (3.12, 7.92)**	** <0.001**	**4.72 (2.26, 7.18)**	** <0.001**	1.57 (−0.78, 3.93)	0.191	0.86 (−2.26, 3.97)	0.590

Model 1: unadjusted.

Model 2: adjusted for health-related factors (number of clinic visits, type of health provider).

Model 3: adjusted for model 2 covariates + age + sex + location (urban/rural).

Propensity-score weighted model: adjusted for health-related factors and chronic conditions (hypertension, diabetes, hyperlipidaemia, kidney disease, and coronary artery disease).

Abbreviations: HbA1c = glycated haemoglobin, SBP = systolic blood pressure, DBP = diastolic blood pressure, LDLc = low density lipoprotein cholesterol, EQ-VAS: European quality of life – visual analogue scale (score range from 0 -100), OR = odds ratio, CI = confidence interval.

Findings were robust to multiple imputation of LDL-C and HbA1c (Table S7), with effect estimates consistent with the primary models.

**Figure 4.**
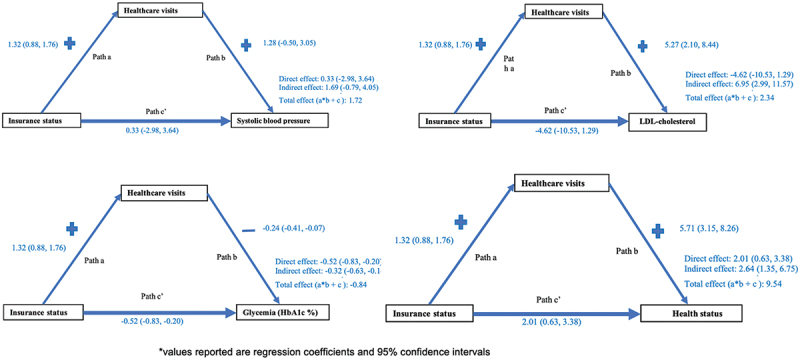
Schematic representation of mediation analysis results using healthcare visits as a mediator.

Table 5 presents mediation analysis results using healthcare visits as a mediator (Figure 4 shows visual representation of mediation analysis results). For mean BP, the indirect effect (1.69, 95% CI: −0.79, 4.05) was larger than the direct effect (0.33, 95% CI: −2.98, 3.64), yielding an RID (ratio of indirect to direct effect) of 5.12, suggesting a strong mediation effect. The RIT (ratio of indirect to total effect) value of 0.98 indicates that most of the total effect of health insurance on BP was explained by the indirect pathway through healthcare visits. For LDLc, both direct (−4.62, 95% CI: −10.53, 1.29) and indirect effects (6.95, 95% CI: 2.99, 11.57) were acting in opposite directions. Specifically, the direct effect was negative (−4.62), while the indirect effect was positive (6.95). Thus, for LDLc, partial mediation with a suppression effect was noted, where the mediator (healthcare visits) enhances or alters the relationship between insurance and LDLc in an unexpected way. For HbA1c, the direct effect (−0.52, 95% CI: −0.83, −0.20) was stronger than the indirect effect (−0.32, 95% CI: −0.63, −0.14), indicating partial mediation. For EQ-VAS (health status), the indirect effect (2.64, 95% CI: 1.35, 6.75) was slightly stronger than the direct effect (2.01, 95% CI: 0.63, 3.38), suggesting partial mediation

**Table 5.** Effect of insurance status and potential mediator (healthcare utilization) on health outcomes.

		Mean BP (mmHg)	Mean LDLc (mg/dl)	Mean HbA1c (%)	Mean EQ-VAS
Mediator	Coefficients	Estimates (95% CI)	Estimates (95% CI)	Estimates (95% CI)	Estimates (95% CI)
Healthcare visit	Direct effect	0.33 (−2.98, 3.64)	−4.62 (−10.53, 1.29)	−0.52 (−0.83, −0.20)	2.01 (0.63, 3.38)
	Indirect effect	1.69 (−0.79, 4.05)	6.95 (2.99, 11.57)	−0.32 (−0.63, −0.14)	2.64 (1.35, 6.75)
	RIT = (Indirect effect/Total effect)	(1.69/1.72) = 0.98	(6.95/2.34) = 2.97	(0.32/0.84) = 0.38	(2.64/9.54) = 0.28
	RID = (Indirect effect/Direct effect)	(1.69/0.33) = 5.12	(6.95/4.62) = 1.50	(0.32/0.52) = 0.62	(2.64/2.01) = 1.31

Abbreviations: HbA1c = glycated haemoglobin, SBP = systolic blood pressure, DBP = diastolic blood pressure, LDLc = low density lipoprotein cholesterol, EQ-VAS: European quality of life – visual analogue scale (score range from 0 -100), OR = odds ratio, CI = confidence interval, RIT, Ratio of indirect effect to total effect; RID, ratio of indirect effect to direct effect.

In sum, these findings indicate varying degrees of mediation by healthcare visits across cardiometabolic outcomes. For blood pressure, the high RIT (0.98) and RID (5.12) suggest that nearly all of the insurance effect was explained by healthcare visits, indicating full mediation. In the LDLc model, the indirect effect via healthcare visits suggests that insurance coverage may increase clinical contact, potentially leading to more frequent initiation or adjustment of lipid-lowering therapy. For HbA1c and EQ-VAS, partial mediation was observed, implying that healthcare visits explain part, but not all, of the observed effects.

## Discussion

Achieving the United Nations’ Sustainable Development Goal of reducing premature NCD deaths by 2030 requires evidence-informed policies the strengthen health systems. This study provides empirical evidence on the relationship between health insurance coverage and cardiometabolic risk profiles among people with chronic conditions in India using a quasi-experimental design. Our findings reveal low insurance coverage among adults with chronic conditions who used health services in the previous year, with fewer than 1 in 15 individuals reporting having any form of health insurance. Although the recent expansion of publicly funded health insurance programs in India have increased health insurance coverage rates, which nonetheless remain low at 18% in urban and 14% in rural settings [26]. Our focus on the association between insurance status and cardiometabolic outcomes remains relevant, particularly given the limited changes in benefit packages for chronic disease care.

Substantial socio-demographic and economic disparities in insurance coverage were observed. Middle-aged to older men, those with advanced education, and higher wealth indices were more likely to be insured, consistent with prior studies from India and other LMICs [46–48]. Gender disparities in coverage may partially explain lower utilization of effective cardiometabolic treatments among women [14,49,50]. Urban-rural differences reflect state-level health insurance programs and target populations at the time of study data collection [51,52]. Public health insurance programs mostly target vulnerable populations, yet their benefit packages often exclude outpatient consultations, medications, and follow-up services NCDs. Fragmented implementation, variable facility empanelment, and bureaucratic barriers further hinder enrolment. Many schemes also exclude informal sector workers, leaving gaps in coverage [53,54]. These systemic constraints limit both uptake and impact of health insurance for chronic conditions, threatening progress toward Universal Health Coverage and NCD control without targeted policy reforms.

Despite limited coverage, bivariate analysis showed insured adults with chronic conditions reported higher health status, and better cardiometabolic risk profiles, consistent with evidence from both high-income countries and LMICs [24,55,56]. Health insurance can reduce financial barriers to quality care, facilitate regular access to outpatient consultations, diagnostics, and medications, which is crucial for asymptomatic conditions like hypertension. Insurance may also engage engagement with the health system and adherence to clinical guidelines, prompting early detection and continued management. Notably, insured participants reported higher smoking prevalence, suggesting potential adverse selection, where individuals at higher health risk are more likely to enroll in health insurance [57,58]. In a low-penetration insurance context such as in this study, adverse selection may influence observed effects, as individuals with greater health needs are more likely to enroll, potentially amplifying associations with healthcare utilization and risk factor management. At the same time, the limited coverage of healthier individuals may attenuate population-level benefits, and these dynamics could interact with provider capacity and incentives, shaping the overall impact of health insurance on cardiometabolic risk factors and health status.

Our findings also show that adults without clinic visits or health insurance were more likely to experience elevated blood pressure, cholesterol and glycemia than those attending at least three clinic visits in the past year. This aligns with prior evidence indicating persistent glycemia control gaps between insured and uninsured populations despite improvements in diabetes care over time [59,60]. In our study, the association between insurance and glycemia (HbA1c, ≥8%) weakened after adjusting for confounders, potentially reflecting limited access to antidiabetic medications, which are costly and often excluded from public insurance packages, unlike low-cost generic medications for hypertension care [14,61,62].

Propensity score-weighted analyses showed a protective effect of health insurance on elevated LDL cholesterol (LDLc > 130 mg/dl) and non-significant positive effects on blood pressure, glycemia, and overall health status. Prior literature on insurance effects is mixed; a systematic review of 68 LMIC studies reported improved access to care (80%), financial risk protection (54.3%) and health status (75%) [24]. Recent Indian survey analysis show minimal health insurance impact on the hypertension care cascade, highlighting additional implementation barriers, including informal payments and socio-cultural factors [63]. Integrating initiatives like Janaushadhi Kendras [64] and Mohalla clinics with access to quality-assured, affordable generic medications could strengthen chronic care accessibility and health outcomes.

Together, evidence from this and prior studies suggests health insurance has direct or indirect protective effects on cardiometabolic outcomes, partially mediated by outpatient visit. Regular clinic visits support preventive care, early detection of complications, and information exchange between patients and providers, improving chronic disease management [65]. Hypertension, often asymptomatic condition, is particularly sensitive to insurance-mediated access. Costs of consultations, tests, and medications likely explain observed differences in mean HbA1c, and LDLc between insured and uninsured, with outpatient care frequency acting as a key mediator. Mediation analysis showed a suppression effect for LDL cholesterol (i.e. negative direct effect and positive total indirect effect mediated through healthcare visits), indicating complex dynamics such as variations in prescribing patterns, adherence, or potential overtreatment, warranting further investigation. Our study findings are consistent with global evidence that confirmed that mere possession of health insurance does not guarantee improved physiological outcomes for LDL-C control, particularly in chronic disease care. Coverage may reduce disparities or improve access to care, but the ‘protective effect’ on LDL-C control itself appears non-causal in these real-world settings [66–69]. Future qualitative research could elucidate how insured individuals navigate care and how these behaviors affect long-term chronic disease management.

### Methodological considerations

This study has several limitations. First, a subset of the CARRS and Solan cohort reporting healthcare utilization in the prior 12 months was analysed, which may underestimate population-level insurance coverage. Second, the cross-sectional design limits causal inference, and adverse selection is relevant in low-penetration insurance settings, with high-risk individuals more likely to enrol. Third, although our primary data were collected between 2010 and 2014, our findings remain policy-relevant. Recent nationally representative data (2018–2023) confirm that overall insurance penetration in India remains low (14% − 18%) and that outpatient and chronic NCD services remain largely excluded from both public and private schemes [9,70]. Fourth, we operationalized insurance as a binary variable (‘any’ vs. ‘none’), which reflects a limitation of the available dataset. However, this is analytically consistent with the reality that most insurance schemes in India during the period studied – whether public or private – offered similarly limited coverage, especially with respect to outpatient chronic NCD care. Thus, the binary categorization captures the essential contrast between having some financial protection versus none, while acknowledging that scheme heterogeneity could not be examined. Lastly, we acknowledge 12% missingness for LDL-C and HbA1c, which can raise concerns about bias. Although baseline characteristics were similar for participants with versus without these measurements, we conducted multiple imputation and found that the direction and magnitude of associations were consistent with the primary findings.

Persistent barriers such as limited outpatient benefits, administrative hurdles, and regional inequities must be addressed to ensure insurance translates into meaningful health gains. Success of programs like PMJAY depends on comprehensive NCD outpatient services.

## Conclusions

This study revealed low health insurance coverage among people with chronic conditions in India. While one in four adults’ lives with a chronic condition, only one in fifteen has any form of health insurance, leading to significantly high out-of-pocket expenditures. Despite limited coverage, insurance status was associated with better cardiometabolic outcomes among adults with chronic conditions in India, largely mediated through increased healthcare utilization. To achieve national and global NCD targets, health insurance schemes must prioritize inclusion of outpatient and preventive services and address equity gaps in access to quality chronic care.

## Supplementary Material

Supplementary Tables_29 09 2025.docx

## Data Availability

KS, DK and DP have access to study data set and statistical code. Any request for data sharing should be addressed to the corresponding author (KS).

## Abbreviations

CARRS: Centre for Cardiometabolic Risk Reduction in South Asia
EQ-VAS: European Quality of Life Visual Analogue Scale
LDLc: Low-Density Lipoprotein Cholesterol
HbA1c: Haemoglobin A1c
BP: Blood Pressure
LMICs: Low- and Middle-Income Countries
NCDs: Non-Communicable Diseases
SDG: Sustainable Development Goal
AB-PMJAY: Ayushman Bharat – Pradhan Mantri Jan Arogya Yojana
RSBY: Rashtriya Swasthya Bima Yojana
RIT: Ratio of Indirect to Total Effect
RID: Ratio of Indirect to Direct Effect
