# Supplementary material for: Does health insurance coverage improve cardiometabolic risk factor levels? Quasi-experimental evidence from India
Source: Glob Health Action. 2025 Oct 14;18(1):2570600. doi: 10.1080/16549716.2025.2570600 (PMC12523458; doi:10.1080/16549716.2025.2570600)
Supplement: Supplementary Tables_29 09 2025.docx [file ZGHA_A_2570600_SM0049.docx]

# **Supplementary Tables**

##

## **Table S1:** Variables and their measurements

| **Variables** | **Measurements** |
| --- | --- |
| **Outcome variable:** |  |
| Blood pressure | 0 = <140/90 mmHg 1 = ≥140/90 mmHg |
| LDL cholesterol | 0 = <130 mg/dl 1 = ≥130 mg/dl |
| Glycated haemoglobin (HbA1c) | 0 = <8% 1 = ≥8% |
| Health status (EQVAS) | Continuous scale (0-100) |
| **Explanatory variables:** |  |
| Insurance | 0 = No 1 = Yes |
| *Healthcare factors* |  |
| Number of clinic visits  (in last 12 months) | 0 = No visit 1 = 1-3 visits 2 = 4 or more visits |
| Type of healthcare provider | 1 = Government 2 = Private 3 = Charitable |
| *Individual level factors* |  |
| Age (years) | 1 = <35  2 = 35-54 3 = ≥55 |
| Sex | 1 = Male  2 = Female |
| Location | 1 = Urban 2 = Rural |
| *Co-morbidity* |  |
| Diabetes | 0 = No 1 = Yes |
| Hypertension | 0 = No 1 = Yes |
| Hyperlipidaemia | 0 = No 1 = Yes |
| Coronary heart disease | 0 = No 1 = Yes |
| Chronic kidney disease | 0 = No 1 = Yes |
| **LDLc: Low-density lipoprotein-cholesterol; HbA1c: Glycated haemoglobin* | |

**Table S2:** Comparison of baseline characteristics between groups having complete outcome measures data vs those having data missing for either BP, LDL, or HbA1c

| N=2926 | **Complete data available, N (%)** | **Missing for either BP, LDL or HbA1c,  N (%)** | **p-value** |
| --- | --- | --- | --- |
|  | **2430** | **496** |  |
| **Age in years, mean (SD)** | 54.7 (11.8) | 53.8 (11.9) | 0.13 |
| <35 years | 84 (3.5%) | 22 (4.4%) | 0.46 |
| 35 - 54 years | 1130 (46.5%) | 236 (47.6%) |  |
| ≥55 years | 1216 (50.0%) | 238 (48.0%) |  |
| **Sex** |  |  |  |
| Male | 1065 (43.8%) | 231 (46.6%) | 0.26 |
| Female | 1365 (56.2%) | 265 (53.4%) |  |
| **Education** |  |  |  |
| Up to primary schooling | 536 (22.1%) | 119 (24.0%) | 0.64 |
| High school to Secondary | 1497 (61.6%) | 297 (59.9%) |  |
| Graduation and above | 397 (16.3%) | 80 (16.1%) |  |
| **Occupation** |  |  |  |
| Not working | 1669 (68.7%) | 294 (59.3%) | <0.001 |
| Semiskilled/Unskilled | 249 (10.2%) | 65 (13.1%) |  |
| Trained/Skilled/White collar | 512 (21.1%) | 137 (27.6%) |  |
| **Marital status** |  |  |  |
| Single | 25 (1.0%) | 9 (1.8%) | 0.17 |
| Married | 2132 (87.7%) | 422 (85.1%) |  |
| Widow/Widower | 264 (10.9%) | 61 (12.3%) |  |
| Separated/Divorced | 9 (0.4%) | 4 (0.8%) |  |
| **Asset Index*** |  |  |  |
| Low | 604 (24.9%) | 149 (30.0%) | 0.053 |
| Medium | 739 (30.4%) | 137 (27.6%) |  |
| High | 1086 (44.7%) | 210 (42.3%) |  |
| **City** |  |  |  |
| Chennai | 966 (39.8%) | 262 (52.8%) | <0.001 |
| Delhi | 834 (34.3%) | 187 (37.7%) |  |
| Solan | 630 (25.9%) | 47 (9.5%) |  |
| *Data are available for all other covariates included in the regression models* | | | |

## **Table S3:** Association between insurance status and cardiometabolic risk factors

| **Model 1** | **BP**≥**140/90 mmHg** | **LDLc** ≥**130 mg/dl** | **HbA1c** ≥7**%** | **HbA1c** ≥**8%** | **EQ-VAS** |
| --- | --- | --- | --- | --- | --- |
|  | **Odds Ratio (95% Confidence Intervals)** | | | | |
| **Insurance** |  |  |  |  |  |
| No | 1 | 1 | 1 | 1 | 1 |
| Yes | 0.85 (0.63,1.14) | 0.83 (0.60,1.15) | 0.70 (0.51, 0.96) | 0.56 (0.39,0.82) | 5.52 (3.12, 7.92) |

##

## **Table S4:** Association between insurance status and cardiometabolic risk factors adjusted for health facility related factors

| **Model 2** | **BP**≥**140/90 mmHg** | **LDLc** ≥**130 mg/dl** | **HbA1c** ≥7**%** | **HbA1c** ≥**8%** | **EQ-VAS** |
| --- | --- | --- | --- | --- | --- |
|  | **Odds Ratio (95% Confidence Intervals)** | | | | |
| **Insurance** |  |  |  |  |  |
| No | 1 | 1 | 1 | 1 | 1 |
| Yes | 0.80 (0.59,1.09) | 0.63 (0.45,0.88) | 0.87 (0.63,1.22) | 0.65 (0.44,0.96) | 4.72 (2.26, 7.18) |
| **Out-patient visits** |  |  |  |  |  |
| No visit | 1 | 1 | 1 | 1 | 1 |
| 1-3 visit | 0.65 (0.27,1.56) | 0.73 (0.28,1.92) | 1.92 (0.71,5.18) | 2.61 (0.75,9.11) | -4.21 (-11.08, 2.66) |
| ≥4 visit | 0.68 (0.29,1.63) | 0.95 (0.36,2.48) | 1.60 (0.59,4.31) | 2.27 (0.65,7.91) | -2.92 (-9.76, 3.93) |
| **Type of health facility** |  |  |  |  |  |
| Government | 1 | 1 | 1 | 1 | 1 |
| Private | 0.89 (0.76,1.05) | 0.64 (0.54,0.76) | 1.56 (1.32,1.85) | 1.36 (1.14,1.62) | -1.25 (-2.51, -0.003) |
| Charitable | 1.80 (0.85,3.82) | 0.49 (0.21,1.17) | 1.69 (0.78,3.63) | 1.09 (0.47,2.51) | 0.87 (-4.93, 6.67) |

##

## **Table S5:** Association between insurance status and cardiometabolic risk factors adjusted for health related factors, age, gender, and location

| **Model 3** | **BP**≥**140/90 mmHg** | **LDLc** ≥**130 mg/dl** | **HbA1c** ≥7**%** | **HbA1c** ≥**8%** | **Health status (EQVAS)** |
| --- | --- | --- | --- | --- | --- |
|  | **Odds Ratio (95% Confidence Intervals)** | | | | |
| **Insurance** |  |  |  |  |  |
| No | 1 | 1 | 1 | 1 | 1 |
| Yes | 0.70 (0.51, 0.96) | 0.56 (0.39, 0.80) | 1.06 (0.75, 1.50) | 0.79 (0.53, 1.18) | 1.57 (-0.78, 3.93) |
| **Out-patient visits** |  |  |  |  |  |
| No visit | 1 | 1 | 1 | 1 | 1 |
| 1-3 visit | 0.66 (0.27, 1.58) | 0.74 (0.28, 1.98) | 2.17 (0.81, 5.84) | 2.75 (0.79, 9.59) | -5.88 (-12.36, 0.61) |
| ≥4 visit | 0.61 (0.25, 1.47) | 0.71 (0.27, 1.88) | 2.26 (0.84, 6.09) | 3.04 (0.88, 10.60) | -6.79 (-13.27, -0.32) |
| **Type of health facility** |  |  |  |  |  |
| Government | 1 | 1 | 1 | 1 | 1 |
| Private | 1.06 (0.89, 1.26) | 0.94 (0.78, 1.15) | 1.15 (0.96, 1.38) | 0.98 (0.81, 1.18) | 2.59 (1.31, 3.87) |
| Charitable | 2.20 (1.03, 4.71) | 0.70 (0.29, 1.71) | 1.16 (0.54, 2.53) | 0.75 (0.32, 1.74) | 5.92 (0.42, 11.43) |
| **Age group** |  |  |  |  |  |
| <35 years | 1 | 1 | 1 | 1 | 1 |
| 35-54 years | 1.70 (1.08, 2.66) | 2.54 (1.44, 4.50) | 2.01 (1.25, 3.22) | 1.69 (1.01, 2.81) | 0.13 (-2.94, 3.21) |
| ≥55years | 2.25 (1.44, 3.53) | 2.98 (1.69, 5.28) | 2.36 (1.47, 3.79) | 1.56 (0.93, 2.60) | -2.89 (-5.97, 0.19) |
| **Sex** |  |  |  |  |  |
| Male | 1 | 1 | 1 | 1 | 1 |
| Female | 0.98 (0.84, 1.14) | 2.02 (1.70, 2.41) | 0.96 (0.81, 1.12) | 0.85 (0.71, 1.01) | -5.06 (-6.20, -3.92) |
| **Location** |  |  |  |  |  |
| Urban | 1 | 1 | 1 | 1 | 1 |
| Rural | 1.55 (1.26, 1.92) | 2.65 (2.11, 3.33) | 0.33 (0.26, 0.41) | 0.31 (0.24, 0.41) | 13.52 (11.94, 15.10) |

# **Abbreviations**: *HbA1c=glycated haemoglobin, SBP=systolic blood pressure, DBP=diastolic blood pressure, LDLc=low density lipoprotein cholesterol, Health status measured using EQ-VAS: European quality of life – visual analogue scale (score range from 0 -100)*

## **Table S6:** Association between insurance status and clinic visits (0-3 & ≥4) adjusted for type of health provider, age, sex, and location

| **Covariates** | **Model 1 (Crude)** | | **Model 2 (type of health provider)** | | **Model 3 (model 2 + age, sex, location)** | |
| --- | --- | --- | --- | --- | --- | --- |
|  | OR  (95% CIs) | p-value | OR  (95% CIs) | p-value | OR  (95% CIs) | p-value |
| **Insurance** |  |  |  |  |  |  |
| No | 1 |  | 1 |  | 1 |  |
| Yes | **3.13**  **(2.13, 4.62)** | **<0.001** | **2.13**  **(1.43, 3.17)** | **<0.001** | **1.54**  **(1.00, 2.36)** | **0.052** |
| **Type of health facility** |  |  |  |  | 1 |  |
| Government |  |  | 1 |  | 1 |  |
| Private |  |  | **0.41 (0.35, 0.48)** | **<0.001** | **0.73 (0.61, 0.87)** | **<0.001** |
| Charitable |  |  | 0.65 (0.31, 1.37) | 0.257 | 1.18 (0.55, 2.51) | 0.677 |
| **Age group** |  |  |  |  |  |  |
| <35 years |  |  |  |  | 1 |  |
| 35-54 years |  |  |  |  | 1.07 (0.70, 1.64) | 0.754 |
| ≥55years |  |  |  |  | 1.32 (0.85, 2.02) | 0.213 |
| **Sex** |  |  |  |  |  |  |
| Male |  |  |  |  | 1 |  |
| Female |  |  |  |  | **1.46 (1.24, 1.72)** | **<0.001** |
| **Location** |  |  |  |  |  |  |
| Urban |  |  |  |  | 1 |  |
| Rural |  |  |  |  | **9.90 (7.06, 13.88)** | **<0.001** |

OR=odds ratio, CIs= confidence intervals

Table S7. Association between insurance status and cardiometabolic risk factors and overall health status - multiple imputation results accounting for missing outcomes data

| **Outcomes** | **Model 1**  (Crude) | | **Model 2**  (adjusted for health-related factors) | | **Model 3**  (adjusted for health-related + Individual factors) | | **Propensity score-weighted model** | |
| --- | --- | --- | --- | --- | --- | --- | --- | --- |
|  | OR  (95% CIs) | p-value | OR  (95% CIs) | p-value | OR  (95% CIs) | p-value | OR  (95% CIs) | p-value |
| **BP ≥140/90 mmHg** | 0.85  (0.63, 1.15) | 0.29 | 0.82  (0.60, 1.11) | 0.19 | **0.71**  **(0.52, 0.97)** | **0.029** | 1.05 (0.64, 1.74) | 0.84 |
| **LDLc ≥130 mg/dl** | 0.83  (0.59, 1.15) | 0.26 | **0.67**  **(0.47, 0.94)** | **0.02** | **0.59**  **(0.41, 0.84)** | **0.004** | **0.49 (0.28, 0.86)** | **0.01** |
| **HbA1c ≥8%** | **0.59**  **(0.41, 0.85)** | **0.004** | **0.68**  **(0.47, 1.00)** | **0.05** | 0.82  (0.55, 1.21) | 0.32 | 1.12 (0.56, 2.22) | 0.76 |
